# Effect of quinolinic acid on human astrocytes morphology and functions: implications in Alzheimer's disease

**DOI:** 10.1186/1742-2094-6-36

**Published:** 2009-12-10

**Authors:** Ka Ka Ting, Bruce J Brew, Gilles J Guillemin

**Affiliations:** 1 St Vincent's Centre for Applied Medical Research, St Vincent's Hospital, Sydney, Australia; 2 Department of Pharmacology, University of New South Wales, Sydney, Australia; 3 Departments of Neurology and HIV Medicine, St Vincent's Hospital, Sydney, Australia

## Abstract

The excitotoxin quinolinic acid (QUIN) is synthesized through the kynurenine pathway (KP) by activated monocyte lineage cells. QUIN is likely to play a role in the pathogenesis of several major neuroinflammatory diseases including Alzheimer's disease (AD). The presence of reactive astrocytes, astrogliosis, increased oxidative stress and inflammatory cytokines are important pathological hallmarks of AD. We assessed the stimulatory effects of QUIN at low physiological to high excitotoxic concentrations in comparison with the cytokines commonly associated with AD including IFN-γ and TNF-α on primary human astrocytes. We found that QUIN induces IL-1β expression, a key mediator in AD pathogenesis, in human astrocytes. We also explored the effect of QUIN on astrocyte morphology and functions. At low concentrations, QUIN treatment induced concomitantly a marked increase in glial fibrillary acid protein levels and reduction in vimentin levels compared to controls; features consistent with astrogliosis. At pathophysiological concentrations QUIN induced a switch between structural protein expressions in a dose dependent manner, increasing VIM and concomitantly decreasing GFAP expression. Glutamine synthetase (GS) activity was used as a functional metabolic test for astrocytes. We found a significant dose-dependent reduction in GS activity following QUIN treatment. All together, this study showed that QUIN is an important factor for astroglial activation, dysregulation and cell death with potential relevance to AD and other neuroinflammatory diseases.

## Background

In physiological conditions, the kynurenine pathway (KP) catabolises the essential amino acid L-tryptophan (L-TRP) to nicotinamide adenine dinucleotide (NAD^+^). During inflammation, the KP can be activated by cytokines and more particularly interferon-γ (IFN-γ) leading to the production of quinolinic acid (QUIN) by monocyte lineage cells. QUIN is an endogenous competitive agonist of the N-methyl-D-aspartate (NMDA) receptor, acting specifically on the subgroup containing the NR2A and NR2B subunits [1,2]. QUIN neurotoxicity has been shown to be involved in the pathogenesis of several age-related neurodegenerative processes associated with neuroinflammation including Alzheimer's disease (AD) [3-6]. Earlier studies with animal models have found that QUIN levels increase with age in the cortex of rats [7]. The neural damage caused by QUIN is similar to the pathologic characteristics of age related-AD [8]. Interestingly QUIN shares several mechanisms with amyloid beta (Aβ) in terms of neurotoxicity and neuroinflammation (Table 1).

**Table 1:** Summary of the effects of QUIN in comparison with Aβ mediated toxicity

QUIN toxicity	References	Aβ toxicity	References
• **Free radical production **via over-activation of NMDA receptor and/or QUIN-Fe2+ complexes and consequent lipid peroxidation and cell death.	(Platenik et al., 2001) (Stone and Perkins, 1981)	• **Free radical production **via Fenton reaction by metals Cu and Fe and consequent lipid peroxidation and apoptosis.	(Huang et al., 1999) (Varadarajan et al., 2001) (Markesbery and Lovell, 1998) (Tamaoka et al., 2000)

• **Excessive PARP activation **leading to NAD depletion.	(Maldonado et al., 2007)	• DNA damage by ROS leads to **PARP over-activation **and NAD depletion.	(Meyer et al., 2006) (Love et al., 1999)

• **Activation of astrocytes **including release of inflammatory chemokines and astrogliosis.	(Guillemin et al., 2003b) (Dihne et al., 2001) (Hanbury et al., 2002)	• **Activation of microglia **and other immune cells leading to secretion of inflammatory cytokines and proteins. • **Co-activation of astrocytes **by inflammatory factors leading to further release of cytokines and astrogliosis.	Griffin and Mrak, 2002) (Murphy et al., 1998) (Selmaj et al., 1990)

• **Inhibition of glutamate uptake **leading to excitotoxicity.	(Tavares *et al*., 2002)	• Aβ can **increase extracellular glutamate **resulting in NMDA receptor over-activation and excitotoxicity.	(Lafon-Cazal et al., 1993; Keller et al., 1997; Lauderback et al., 2001) (Harris et al., 1995; Harris et al., 1996)

• NMDA receptor activation by QUIN can lead to Aβ production.	(Lesne *et al*., 2005)	• Aβ can induce IDO in the KP and increase production of QUIN.	(Guillemin et al., 2003a)

Alterations of the functionality of glial cells, including changes in morphology and proliferative activity, are a common feature of neuroinflammation [9]. Microglia represent the major immune cell type within the brain. When activated, they target foreign molecules including amyloid plaques, and secrete cytokines, free radicals, and other cytotoxic substances that rapidly become neurotoxic [10]. Activated microglia are the major source of QUIN during brain inflammation [11]. We previously showed that activated microglia surrounding the amyloid plaque are highly immunoreactive for QUIN [5]. Astrocytes are also involved in the glial response during neuroinflammation and closely interact with microglia [12]. The presence of reactive astrocytes is a known feature of AD pathology. Astrogliosis with increased vimentin (VIM) and decreased glial fibrillary acidic protein (GFAP) expression, and marked elevations in inflammatory, immune, and oxidative stress markers, extracellular matrix molecules, and cytokines are also common features of AD [13,14]. We previously showed that QUIN up-regulates chemokine production and chemokine receptor expression in primary human astrocytes [15]. Moreover, low doses of QUIN or IL-1β alone do not have significant deleterious effects but in combination lead to a significant loss of pyramidal neurons in the rat hippocampus [16]. However, it still remains unknown whether QUIN production from Aβ-activated microglia can directly induce production of pro-inflammatory cytokines by astrocytes. We previously demonstrated that 500 and 1200 nM QUIN induces apoptosis in human foetal astrocytes (10 and 15%, respectively) [17]. To our knowledge, the effects of QUIN on astrocyte proliferation, cytokine production and enzymatic functions have not been studied. Moreover, it is unknown whether low sub-toxic doses of QUIN would potentially have proliferative and/or apoptotic effects on human astrocytes. To further examine this, we focused on the expression of cytoskeleton proteins (GFAP and VIM) and astrocyte proliferation as it is plausible that in addition to apoptosis by QUIN, the remaining 85 to 95% of live astrocytes may undergo astrogliosis (hypertrophy) or astrocytosis (hypertrophy and proliferation). Additionally, neuronal activity leads to production of glutamate, which is then taken up by astrocytes, via Na^+ ^-dependent glutamate transporters, and recycled to glutamine [18]. The regulatory enzyme of the glutamate-glutamine cycle in astrocytes is glutamine synthetase (GS). GS inhibition has been previously described in AD pathogenesis [19].

The present study was designed to determine: 1) the effect of QUIN on human primary astrocytes in the expression of AD-related pro-inflammatory cytokines, 2) the ability of QUIN to activate astrocytes and induce their proliferation at lower doses and 3) to investigate the effect of increasing doses of QUIN on GS activity as a functionality test. Understanding the effects of QUIN on human astrocytes is essential to better understand the neuroinflammatory mechanisms involved in AD and other neuroinflammatory diseases and to facilitate the development of new therapeutic targets for neurodegenerative diseases [20].

## Materials and methods

### Reagents

#### Cell culture

Dulbecco's phosphate buffered saline (PBS) 1×, RPMI medium 1640 1×, 0.5% Trypsin-EDTA 10×, Glutamax-1 100×, Antibiotic-Antimycotic (AA) 100× were obtained from GIBCO Invitrogen (Victoria, Australia). Glucose intravenous infusion BP 50% was from AstraZeneca (Sydney, Australia). Foetal calf serum (FCS) was obtained from Bovogen Australia. Culture flasks and plates were purchased from Becton Dickinson labware (New Jersey, USA). Recombinant human IFN-γ, recombinant TNF-α and TGF-α were from RnD Systems. Quinolinic acid (QUIN) was obtained from Sigma Chemical (Sydney, Australia).

#### PCR and electrophoresis

Rneasy Mini Kit and Q solution were obtained from QIAGEN (Victoria, Australia). Buffer 10× 1.5 mM, Taq polymerase, dNTP 10 mM were obtained from Roche (Sydney, Australia). Accuprime PCR system and cyto-9 fluorescence dye were purchased from Invitrogen. PCR primers were purchased from Geneworks (South Australia, Australia). Agarose 1 biotechnology grade powder was obtained from Amresco (Ohio, USA).

##### End Point PCR reaction per tube (100 μL)

10 μL Buffer 10× pre-mixed with MgCl_2 _1.5 mM + 2.5 μL dNTP 10 mM + 1 μL (10 μM) Forward primer + 1 μL (10 μM) Reverse primer + 0.5 μL Taq Polymerase + 80 μL DEPC H_2_O + 5 μL sample cDNA.

##### Real-time PCR reaction per tube (20 μL)

2 μL Buffer 10× pre-mixed with MgCl_2 _3 mM + 0.4 μL dNTP 10 mM+3 μL Q solution + 0.2 μL SybrGreen (1:1000), 0.2 μL Taq Polymerase + 0.2 μL (10 μM) forward and reverse primer + 9 μL H_2_O + 5 μL diluted sample cDNA (1 part stock cDNA in 4 parts of nuclease-free H_2_O)

#### GFAP and vimentin enzyme linked immunosorbent assay (ELISA)

Human GFAP ELISA kit was obtained from Biovendor Laboratory Medicine, Inc. (Modrice, Czech Republic). As there are no commercial ELISA kits for vimentin, an indirect vimentin ELISA protocol was generated. Primary monoclonal mouse vimentin antibody (0.5 mg/mL) used in the ELISA was purchased from BD Pharmingen (California, USA). Secondary goat anti-mouse antibodies conjugated with horseradish peroxidise (0.5 mg) were obtained from Novus Biologicals (Littleton, USA). The coating buffer consisted of 3.03 g Na_2_CO_3_, 6 g NaHCO_3 _from Lancaster (Morecambe, England) in 1 litre (L) dH_2_O (pH 9.6). 3,3'5, 5'-Tetramethylbenzidine (TMB) liquid substrate system for ELISA (ready to use), bovine serum albumin (BSA) (pH 7), and 0.05% PBS-tween (PBS-T) were purchased from Sigma Aldrich (Sydney, Australia). Wash solution was made up of 0.05% PBS-tween. Stop solution composed of 2 N sulfuric acid (H_2_SO_4_) was used in both GFAP and vimentin ELISA. NUNC maxisorp flat bottom ELISA plates (New York, USA) were used for the vimentin ELISA study. Samples were sonicated in PBS supplemented with 1× EDTA-free "complete" cocktail of protease inhibitors purchased from Roche Diagnostics (Mannheim, Germany).

#### MTT (3-(4,5-dimethylthiazol-2-yl)-2,5-diphenyltetrazolium bromide) assay

MTT was obtained from Sigma Chemical. Sodium dodecyl sulfate (SDS) was sourced from International Biotechnologies Inc (Connecticut, USA) and *N, N*-dimethylformamide (DMF) from Ajax Chemicals (Auburn, Australia).

#### Glutamine synthetase activity assay

##### Lysis buffer

HEPES (20 mM), NaCl (118 mM), KCl (4.6 mM), KH_2_PO_4 _(1.1 mM), MgCl_2 _(1.2 mM) and 1× Complete-EDTA free inhibitors.

##### Reaction buffer

Imidazole-HCl (50 mM, pH 7.2), KH_2_AsO_4 _(25 mM), hydroxylamine/NH_2_OH (50 mM), **L-glutamine (50 mM), MnCl_2 _(0.5 mM), Adenosine 5'-diphosphate sodium salt (ADP) (0.4 mM).**

##### Stop solution

FeCl_3 _(0.37 M), hydrochloric acid (HCl) (0.67 M) and trichloroacetic acid (TCA) (0.2 M).

##### Chemicals

HEPES from JRH Biosciences (Kansas, USA), NaCl from Merck, KCl, hydroxylamine/NH_2_OH, L-glutamine, ADP, FeCl_3 _and KH_2_AsO_4 _from Sigma Chemicals (Sydney, Australia), KH_2_PO_4_, MgCl_2 _from Ajax Chemicals and 1× Complete-EDTA free inhibitors from Roche Diagnostics. Imidazole-HCL from Aldrich Chemical Company, Inc. (Milwaukee, USA), Tri-Sodium Citrate, HCl and TCA from Ajax Chemicals and MnCl_2 _from BDH Chemicals Ltd (Poole, England). GS activity was measured using the Cary 50BIO UV spectrophotometer (Varian, Sydney).

#### Cell culture conditions and cell lines

All cells were maintained at 37°C under 5% CO_2 _in humidified air. U251 glioblastoma is a malignant cell line derived from human glia and was obtained from American Type Culture Collection (ATCC). It was used as a positive control for vimentin and GFAP expression and was cultured in Dulbecco's RPMI medium 1640 supplemented with 10% FCS, 1% glutamax-1, 1% AA and 0.5% glucose.

## Methods

### Isolation and culture of primary astrocytes

Human foetal brains were obtained from 18- to 19-week-old foetuses following informed consent. Astrocytes were prepared using a previously described protocol [21]. Briefly, cerebral portions were washed thoroughly with phosphate-buffered saline (PBS), forced through a 100 μm nylon mesh with the plunger of a plastic syringe. The suspension was centrifuged at 500 g for 5 minutes and the cell pellet resuspended in RPMI 1640 medium containing 10% heat-inactivated FCS, 1% glutamax-1, 1% antibiotic-antimicrobial liquid, 0.5% glucose, then plated onto 75 cm^2 ^culture flasks and incubated at 37°C. Medium was changed on the 3^rd^, 5^th ^and 10^th ^day. The cells became confluent after 10-12 days. Microglia were detached from the cultures by mechanically shaking the flasks for 2 hours at 220 rpm at room temperature and aspirated. The astrocytes were passaged at least three times to further purify and isolate astrocytes from contaminating microglia and neurons. Astrocytes were left to recover for 3 days after each passage. The astrocytes were rinsed twice with PBS and cultured as above in uncoated flasks with the culture medium and maintained for up to 6 weeks. The medium was changed twice a week. Culture purity was determined by immunofluorescence analysis with antibodies against GFAP and more than 95% of the cells stained positive for GFAP [22].

Purified astrocytes were subcultured into 12-well plates and left to recover for 48 hours. For the real-time PCR experiments, the astrocytes were treated with IFN-γ (100 IU/mL), TNF-α (100 IU/mL), TGF-α (20 ng/μL) and QUIN (50, 150, 350, 500 and 1200 nM) and incubated for 24 hr at 37°C unless otherwise stated.

### RNA extraction and first strand cDNA synthesis

Astrocytes were washed twice with PBS before extraction. RNA extraction was performed based on the protocol stated by QIAGEN for Rneasy mini kit. Each RNA stock sample was diluted 1:10 and mixed thoroughly, spun down and kept on ice. An RNA standard derived from liver tissue (50 ng/μL) was diluted 1:10 to make standards of 10, 20, 30, 40, 50, 60 ng. Each diluted RNA sample was added into 96-well plates at volumes of 3 μL and 7 μL. Next, a stock solution of TE buffer (10×) was diluted to 1× and 0.1 μL SYBRgreen II dye was added to each mL of TE buffer. Finally, 0.2 mL of 1× TE-SYBRgreen II buffer was added into each well of standard and samples. Blank wells contained no samples nor standards, only 1× TE-SYBRgreen II buffer. Fluorescence intensity for the standard and the samples was measured using FLUOstar Optima fluorometer machine (BMG Labtech, Victoria, Australia). A standard curve was formed and RNA concentration was determined from the standard curve equation. cDNA synthesis was performed based on the protocol stated by Invitrogen for RT-PCR system using ThermoScript™. For each sample, 0.5 μg RNA was used for the cDNA synthesis.

#### End-point and real-time PCR

End-point PCR conditions for all primers were: denaturation at 94°C for 1.5 minutes, annealing at 60°C for 30 seconds, followed by extension at 72°C for 60 seconds. All end-point PCR was done in 35 cycles using an Eppendorf thermocycler. The purpose of end-point PCR was to screen semi-quantitatively for the presence of the following genes in primary human foetal astrocytes.

Standards of cDNA for β-actin, vimentin, GFAP and IL-1β to be used for quantitative measure in real-time PCR were synthesized using the double end-point PCR method that utilizes real-time PCR conditions. Briefly, 5 μL of stock cDNA for each gene of interest was mixed with 15 μL master mix in duplicates and end-point PCR of 45 cycles was performed. PCR products were analysed on the agarose gel for correct product size. Next, add 5 μL of 1^st ^PCR aliquot to 15 μL of 2^nd ^PCR master mix in duplicates and a 2^nd ^end-point PCR is performed with the same condition and cycles as the 1^st ^end-point PCR. At this step, both duplicate tubes for each standard are mixed together to make a total volume of 40 μL and loaded on to agarose gel for electrophoresis. The product bands for each standard should be significantly thicker when viewed with UV illuminator. The product bands for each standard are then cut and extracted using QIAGEN gel extraction kit according to manufacturer's protocol. Each PCR standard concentration was measured using the previously described method for determining RNA concentration with the exception of using SybrGreen dye I which is specific to detect cDNA. After measuring the concentration, the standards were diluted to a stock concentration of 10 ng/μL. Before performing the real-time PCR, the standards were serial diluted from the stock 1 in 10. Finally, the standards were mixed with the master mix that was used for the samples and loaded into the machine to amplify. A standard curve can be generated from the values given and the unknown concentration of each amplified sample can be determined.

Table 2 shows the PCR primer sequences used for real-time PCR. PCR conditions for β-actin, vimentin and IL-1β: denaturation (HOLD) at 95°C for 5 minutes then denaturation starts at 95°C for 10 seconds, annealing at 60°C for 15 seconds, extension at 72°C for 15 seconds, followed by a melt up to 99°C. β-Actin, vimentin and IL-1β real-time PCR was done in 40-45 cycles. PCR conditions for GFAP: denaturation (HOLD) at 94°C for 3 minutes then denaturation starts at 94°C for 10 seconds, annealing at 58°C for 15 seconds, extension at 68°C for 30 seconds, followed by a melt up to 99°C. GFAP real-time PCR was done in 40 cycles. All real-time PCR was carried out in triplicates using Corbett Lightcycler, *n *= 3. Concentrations of each samples and controls for β-Actin, vimentin, GFAP and IL-1β were calculated from their respective standard curve. Subsequently, we normalized the concentration values of samples and controls found for vimentin, GFAP and IL-1β with their respective β-Actin values to rule out any differences in load between samples and controls. Normalized untreated controls are then calculated as 100% and the percentage of samples was calculated relative to their controls. For example, a sample percentage of 180 would be calculated as 80% increase above controls. After calculating the percentages of each samples and controls, these percentages (in triplicates) are put together with the values of 2 other triplicates derived from 2 independent PCRs and the standard deviation and p-values are determined using PRISM.

**Table 2:** PCR primer sequences

End-point PCR Primers	Forward	Reverse	Size (bp)
GAPDH	ACCACCATGGAGAAGGCTGG	CTCAGTGTAGCCCAGGATGC	509

IL-1β	ATG GCAGAAGTACCTGAGCTC	TTAGGAAGACACAAATTGCATGGTGAA	810

IL-6	GTGTGAAAGCAGCAAAGAGGC	CTGGAGGTACTCTAGGTATAC	159

GFAP	CTGGGCTCAAGCAGTCTACC	CTGGGGTTAAGAAGCAGCAG	666

GS	AAGTGTGTGGAAGAGTTGCC	TGCTCACCATGTCCATTATC	234

S100β	ATGTCTGAGCTGGAGAAGG	CTGTCTGCTTTCTTGCATG	415

**Real-time PCR Primers**	**Forward**	**Reverse**	**Size (bp)**

β-actin	TCACCCACACTGTGCCCATCTACGA	CAGCGGAACCGCTCATTGCCAATGG	295

Vimentin	GAGAACTTTGCCGTTGAAGC	TCCAGCAGCTTCCTGTAGGT	170

IL1β	AAGGCGGCCAGGATATAACT	CCCTAGGGATTGAGTCCACA	102

GFAP	TCTCTCGGAGTATCTGGGAACTG	TTCCCTTTCCTGTCTGAGTCTCA	81

### Electrophoresis

All PCR products were then loaded into 2% Agarose gels supplemented with 0.001% ethidium bromide (EtBr). Gel electrophoresis was carried out at 100 V for 40 minutes and analysed using Syngene UV illuminator machine.

### GFAP ELISA

Human foetal astrocytes were plated onto 12-well plates and grown until 90% confluent in RPMI with 10% FCS overnight. The cells were stimulated with IFN-γ, TNF-α, TGF-α and QUIN at 50, 150, 350, 500 and 1200 nM for 24 hrs. Cells were harvested by Trypsin/EDTA, centrifuged at 13,000 rpm, aspirated and re-suspended in PBS. After that, the cells were centrifuged at the same speed to form a pellet at the bottom of the Eppendorf tube and sonicated to lyse the cells. The final volume of each sample was 300 μL. ELISAs for GFAP were carried out according to Biovendor human GFAP ELISA kit protocol. First, prepare human GFAP standards according to manufacturer's dilutions for duplicates. Next, dilute 100 μL of each samples with 200 μL dilution buffer provided by the kit. 100 μL of each standard and samples were added in duplicates and incubated for 2 hours at room temperature. Next, each well was washed 3 times with 350 μL wash solution and after the final wash; the plate was inverted and tapped strongly on paper towel. 100-μL biotin labelled anti-human GFAP polyclonal antibody was added into each well. Immunoreaction was allowed to proceed for 1 hour at room temperature. The plates were then washed 3 times as before and 100 μL streptavidin-horseradish peroxidase conjugate was added to each well and incubated for 1 hour at room temperature. The plates were washed as previously stated followed by the addition of 100 μL tetramethylbenzidine/hydrogen peroxide substrate solution and incubated for 30 minutes covered with foil. The colour development was stopped with stop solution and the absorbance was read at 450 nm.

### Direct vimentin ELISA

The direct vimentin ELISA method was modified from Zeisberg et al [23]. Human foetal astrocytes were cultured and stimulated as described above. Cells were harvested by Trypsin/EDTA, centrifuged at 13,000 rpm, aspirated and re-suspended in PBS. After that, the cells were centrifuged at the same speed to form a pellet at the bottom of the Eppendorf tube and sonicated to lyse the cells. Standards were performed in duplicates and include using U251 cells at different cell densities 8 × 10^5^, 7 × 10^5^, 6 × 10^5^, 5 × 10^5^, 4 × 10^5^, 3 × 10^5^, 2 × 10^5^, 1 × 10^5 ^per well. ELISA plates were coated overnight (4°C) with a constant cell density of 4 × 10^5 ^cell lysates or standards in coating buffer. Wells were aspirated and washed 3 times with 300 μL wash buffer per well. After the last wash, the plate was inverted and blotted on absorbent paper to remove residual buffer. Plates were blocked with 200 μL blocking solution (1% BSA in PBS) per well and incubated at room temperature for 1 hour. Each well was aspirated and washed 3 times with 300 μL wash buffer per well. 100 μL of monoclonal mouse Vimentin primary antibody (1:500) diluted in incubation buffer was added into each well and incubated for 1 hour at room temperature. After primary incubation, each well was aspirated and washed with 300 μL wash buffer 5 times. 100 μL whole mouse IgG HRP conjugated secondary antibody (1:1000) in incubation buffer was added into each well and incubated for 1 hour at room temperature. Next, all the wells were aspirated and washed 5 times. Then into each well, 100 μL of TMB was added as substrate for HRP resulting in a blue colour formation in each well over a 30 minutes incubation period. Finally, adding 100 μL stop solution into each well stopped the reaction resulting in yellow colour development. The plate was measured using an ELISA microplate reader at a wavelength of 450 nm. Sample and standard absorbance were corrected with blank absorbance. VIM protein levels were estimated as a relative value from the standard curve and graphed as a percentage of controls.

### MTT cell viability/proliferation assay

Human foetal astrocytes were subcultured into 96-well plates with a density of 5 × 10^3 ^cells per well. Serum-free, 5% FBS and 10% FBS supplemented normal RPMI 1640 medium were used as negative, normal and positive controls, respectively to maintain astrocyte culture. TGF-α was omitted, as 10% FBS was used as positive control in this assay, even though TGF-α is a known inducer of GFAP expression and astrogliosis [24]. IFN-γ, TNF-α (100 IU/mL) and QUIN at 50, 150, 350, 500 and 1200 nM were added to the astrocytes in 5% FBS supplemented normal RPMI medium. The astrocytes were incubated for 24, 48, 72 and 96 hours. This MTT assay is a modified version from the original protocol by [25]. This colorimetric assay is based on the ability of a mitochondrial dehydrogenase enzyme from viable cells to cleave the tetrazolium rings of the pale yellow MTT and form dark blue formazan crystals which are largely impermeable to cell membranes, thus resulting in their accumulation within healthy cells. At each time point, 20 μL of stock MTT (5 mg/mL) was added into each well containing 0.4 mL of appropriate medium and incubated for 4 hours at 37°C. Next, all media was aspirated from each well and 20% SDS in 50% DMF was added into all wells to solubilise the dark blue formazan crystal product overnight at 37°C. The plates were measured using BioRad Multiskan spectrometer at a wavelength of 560 nm and 620 nm as reference. The number of surviving cells is directly proportional to the level of the formazan product created.

### Glutamine Synthetase (GS) activity in "transferase" reaction

ADP+L-Glutamine+NH_2_OH→γ-glutamyl hydroxamate

The GS transferase reaction assay was adapted from Thorndike and Reif-Lehrer [26]. Human foetal astrocytes were seeded into 12-well plates until confluent with a final density of 5 × 10^3 ^cells per well. The cells were then stimulated with appropriate concentrations of QUIN and incubated for 24 hours at 37°C. Afterwards, the cells were rinsed twice with ice cold PBS. Next, 0.35 mL lysis buffer was added to each well and sonicated and the lysates were collected in Eppendorf tubes. The lysates were centrifuged at 13,000 rpm for 5 minutes and supernatants transferred to new Eppendorf tubes. All samples were kept on ice at all times until the start of assay. Then, 0.1 mL of each sample supernatant was mixed with 0.4 mL of dH2O and 0.5 mL of reaction buffer to make a final volume of 1 mL in a UV glass cuvette. The enzyme reaction and kinetics were measured at 37°C for 15 minutes and the absorbance was taken at wavelength of 340 nm at 0.1 seconds interval using a Cary 50BIO UV spectrophotometer. GS activity was measured only in the linear region of the kinetic curve and calculated as Δ absorbance per minute. Changes in the rate of GS activity between controls and samples were calculated and graphed in percentage values.

### Statistical analysis

Each experiment was repeated 3 independent times which, included triplicates of each sample and controls. All data from each experimental study was analysed using the statistics software PRISM and unpaired student's t-test was used to compare each treated samples with the untreated controls. *P *values of less than 0.05 (*p *< 0.05) was taken as significant.

## Results

### End-point mRNA expression of pro-inflammatory cytokines and intermediate filaments in human astrocytes

**Figure 1:**
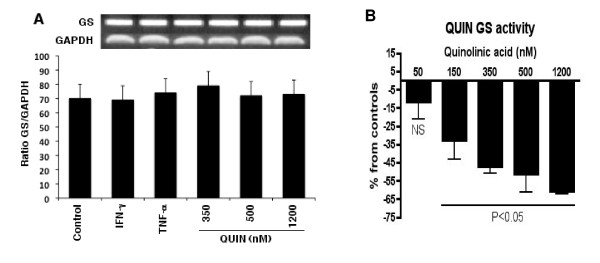
**(A) Ethidium bromide-stained gel showing mRNA expression of GS and GAPDH 24 hrs after cytokine or QUIN treatments**. Results are expressed as a ratio of specific gene expression normalized against expression of the housekeeping gene GAPDH. Standard errors were always 5%. **(B)** Glutamine synthetase activity after 24 hours stimulation with QUIN. GS enzyme assay has been done in triplicate (P < 0.05 was taken as significant).

We used end-point PCR as a basic screening tool to validate the expression of GFAP in primary human cultures of astrocytes. In addition, TNF-α samples showed an increase in IL-1β mRNA expression. GS mRNA expression was constitutively expressed in astrocytes even after stimulation with cytokines and QUIN (Figure 1A). Our data also indicates that astrocytes constitutively expressed IL-6 and S100β (data not shown).

### Real-time mRNA expression of pro-inflammatory cytokines and intermediate filaments in human astrocytes

**Figure 2:**
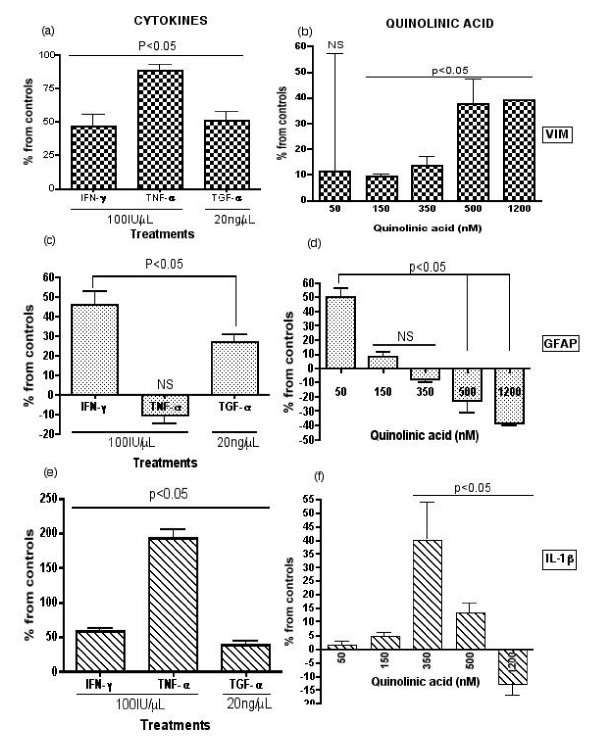
**Expression of VIM, GFAP and IL-1β genes following 24 hours stimulation with cytokine/growth factor and QUIN**. Real-time PCR results for VIM (a and b), GFAP (c and d), IL-1β (e and f). Each experiment repeated, *n *= 3. A statistical value of P < 0.05 was taken as significant.

We performed real-time PCR to quantitatively measure the gene expression of selected genes including vimentin. Real-time PCR results are shown in Figure 2 for Vimentin (a) and (b), GFAP (c) and (d), IL-1β (e) and (f). Vimentin mRNA levels were increased approximately 0.5 to 1-fold by IFN-γ, TNF-α and TGF-α compared to untreated controls. With the exception of QUIN 50 nM, VIM expression was significantly increased by all doses of QUIN. At pathophysiological concentrations QUIN 350, 500 and 1200 nM [4,27,28], VIM expression was respectively increased by 13%, 38% and 40%. Similarly, GFAP expression was increased by cytokines IFN-γ and TGF-α but there was no significant effect with TNF-α. GFAP expression was increased respectively by 49% and 8% with physiological (50 nM) and supra-physiological (150 nM) concentrations of QUIN. In contrast to VIM expression, GFAP expression was gradually reduced with increasing concentrations of QUIN. Treatment with pathophysiological concentrations of QUIN 350, 500 and 1200 nM led respectively to a 9%, 22% and 39% decrease in GFAP expression. IL-1β expression was highest with TNF-α (positive controls). Both IFN-γ and TGF-α up regulated IL-1β expression by 50% and 25%, respectively. IL-1β mRNA reached their highest level of expression in astrocytes treated with QUIN 350 nM but the levels gradually decreased with higher QUIN doses. The levels of IL-1β mRNA induction by QUIN 350 nM were comparable to those induced by IFN-γ.

### ELISA quantification of GFAP and vimentin protein levels in human astrocytes

**Figure 3:**
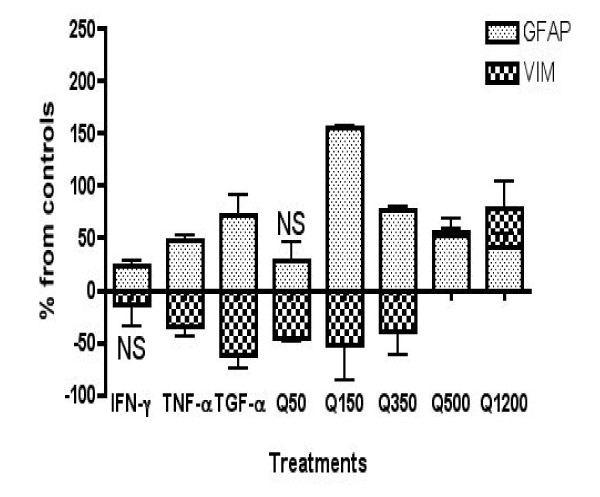
**Quantification of GFAP and vimentin using ELISA after 24 hours stimulation with IFN-γ, TNF-α, TGF-α and QUIN**. GFAP and VIM indirect ELISA have been done in triplicates. All samples except IFN-γ stimulated VIM protein expression and 50 nM QUIN stimulated GFAP expression were statistically significant (p < 0.05).

GFAP and Vimentin sandwich ELISAs were performed in triplicates (Figure 3). All samples except IFN-γ stimulated Vimentin protein expression and QUIN 50 nM stimulated GFAP expression (p < 0.05). There was an increase in GFAP protein levels of 25%, 50% and 75% in samples stimulated with cytokines IFN-γ, TNF-α and TGF-α, respectively. In contrast to real-time PCR data (Figure 2), all concentrations of QUIN except for 50 nM, led to dose-dependent decrease in GFAP expression although GFAP levels were markedly above untreated astrocytes. Similarly with mRNA expression, production of GFAP protein was dose dependently reduced with pathophysiological concentrations of QUIN. Production of VIM protein was opposite to the expression of GFAP with a reduction of 5%, 35% and 55% in samples stimulated with IFN-γ, TNF-α and TGF-α, respectively. Treatments with physiological (50 nM) and supra-physiological (150 nM) concentrations of QUIN led to a 52% and 46% decrease in VIM production respectively. However, with pathological concentrations of QUIN (500 and 1200 nM) VIM protein levels were significantly increased by 58% and 81% respectively compared to untreated controls.

### MTT cell viability/proliferation assay

**Figure 4:**
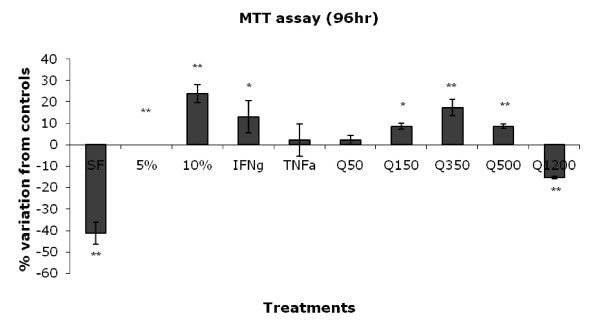
**Proliferation measurement after stimulation with QUIN for 96 hours, SF = serum free, 5% FCS = normal control and 10% FCS = positive control**. Statistical values of P < 0.05 = *, P < 0.01 = ** and P < 0.001 = *** were taken as significant. Proliferation assay was optimized at 96 hours and was done in triplicates. The normal control (5% FCS) was set to 0%.

Astrocytes were left to proliferate for 96 hours. The assay was done in triplicate. The normal control (5% FCS) was set to 100%. As seen in figure 4, TNF-α and samples treated with QUIN 50 nM had no significant effect on astrocyte proliferation. In contrast, IFN-γ had an increase of nearly 10% in cell proliferation. A significant increase in proliferation was observed for samples treated with QUIN 150, 350 and 500 nM. Samples stimulated with QUIN 1200 nM had approximately 10% reduction indicating cell death/apoptosis.

### GS enzyme activity

We found that treatment with QUIN led to a dose dependent decrease in GS enzyme activity (Figure 1B). All samples excluding samples treated with QUIN 50 nM were statistically significant (*p *< 0.05).

## Discussion

Several studies have shown that the levels of the excitotoxin QUIN can reach μM concentrations in the brain parenchyma and CSF during neuroinflammation [4,29]. We previously showed that QUIN is toxic to human neurons and astrocytes at concentrations as low as 100 nM [30,31] and 350 nM [17]. We also previously demonstrated that QUIN is able to activate both adult simian and human foetal astrocytes to a similar extent leading to production of inflammatory chemokines [15]. In the present study, we assessed the effects of QUIN on the level of expression of IL-1β and structural proteins GFAP and VIM, on cell proliferation and on glutamine synthetase activity in primary cultures of human astrocytes.

QUIN at submicromolar concentrations can produce neurotoxicity in culture if maintained for several weeks and neuronal apoptosis upon exposure to 100 nM QUIN [32]. We have chosen the concentrations of QUIN (50, 150, 350, 500 and 1200 nM) in accordance to the concentrations used by previous studies [4,27,33,34]. For the purpose of our study, it is important to use pure cultures of astrocytes to maintain accuracy in measuring astrocytic gene expression, proliferation and GS activity. Due to the overlapping cytokine expression of microglia and astrocytes, our study utilizes only pure cultures of astrocytes to avoid inaccurate quantitative measurement of pro-inflammatory cytokines induced by QUIN. Also, for proliferative studies on astrocytes, it is crucial to use pure cultures of astrocytes to accurately measure proliferation because microglia have been shown to proliferate during activation as well. However, microglia activation and recruitment but no proliferation was sufficient to mediate neurodegeneration [35].

The cytokine IL-1β is known to play important roles in both immune regulation and pathogenesis of AD [36]. Within the CNS, IL-1β is produced by astrocytes, microglia and brain microvascular endothelial cells [37]. IL-1β is known to be over-produced in AD brain and is associated with neuroinflammation, increased Aβ production, deposition and plaque formation [38]. Using real-time PCR, we found that at low concentrations QUIN induces a dose dependent increase in IL-1β mRNA level and would likely increase IL-1β production and secretion. However, we could not detect any significant IL-1β production in the culture supernatant using ELISA (data not shown). There are several potential explanations for these results. The first is that damaged astrocytes undergoing necrosis release the inactive pro-forms of IL-1β whereas apoptotic astroglial cells produce the mature form of IL-1β [39]. We previously showed that only a small proportion of astrocytes undergo apoptosis following treatment with QUIN 500 and 1200 nM [40], therefore the level of mature IL-1β produced and released is likely to be low and under the level of detection. Secondly, other studies have reported that co-culture of microglia activated with LPS, a potent inducer of QUIN production, with astrocytes leads to significantly higher astroglial IL-1β production compared to cultures with inactivated microglia. These data suggest that microglial cells play a key role in IL-1β production by astrocytes [11,41]. Interestingly, at low concentrations neither QUIN (80 and 120 nM) nor IL-1β (10 ng/μL) separately induced any neuronal damage, but in combination lead to a significant loss of pyramidal neurons in rat hippocampus [16]. This synergetic action is likely to play a significant role in the neuropathogenesis of AD, as both molecules are concomitantly present in the AD brain.

Cytoskeleton re-organization is an important step of the astroglial response to pathologic stimuli [42,43]. Astrocyte activation (astrogliosis) is characterized by changes in co-existing intermediate filaments, particularly GFAP and VIM, together with cellular hypertrophy and/or hyperplasia [44]. In physiological conditions, human astrocytes in the white matter are weakly immunoreactive for VIM but in inflammatory areas in AD brain over-express VIM [45]. Our real-time PCR results (Figure 2a) show that IFN-γ, TNF-α and TGF-α can significantly increase VIM mRNA expression compared to untreated controls. Izmailova et al. previously showed that IFN-γ also induces mRNA expression of VIM in HeLa cells [46], which is in accordance with our data. However, our ELISA results showed that treatment with IFN-γ, TNF-α and TGF-α led to a reduction in levels of VIM protein compared to untreated controls. These results suggest that VIM expression is likely to be regulated by post-transcriptional or post-translational mechanisms. Several other studies have shown that TNF-α and TGF-α can activate murine astrocytes and induce both VIM and GFAP over-expression [24,47,48]. TGF-α was included in the ELISAs as a positive control, as it is known to induce GFAP expression and astrogliosis [24]. We found that GFAP mRNA expression was significantly increased by IFN-γ and TGF-α but not by TNF-α. ELISA results showed that all three cytokines could induce significant increase in GFAP. Previous studies have found that IFN-γ can induce GFAP mRNA and protein expression in both human and rodent primary astrocytes, which is in accordance with our data [49]. We also found that treatment with neuropathological concentrations of QUIN (>150 nM) leads to a switch between structural protein expressions in a dose dependant manner. QUIN increases VIM and concomitantly decreases GFAP expression at both mRNA and protein levels (Figures 2b and 3) compared to untreated controls. The effect of QUIN is likely to be mediated by the activation of NMDA receptors. Previous studies have shown that NMDA-induced excitotoxicity resulted in increased VIM expression in rat astrocytes [50,51]. Using calcium flux imaging, we recently demonstrated that QUIN is able to activate human foetal astrocytes through functional glial type NMDA receptors (data not shown). We have demonstrated that pathophysiological concentrations of QUIN induce a substantial VIM/GFAP cytoskeleton re-organization, known to be associated with hypertrophy and/or astrogliosis, in human primary astrocytes. The relevance of these data is that QUIN induced-astrogliosis is likely to contribute to scar formation suppressing local axonal regeneration or neuronal regrowth. Using knockout mice for VIM and/or GFAP, Ribotta et al. have found a significant decrease in glial scar formation associated with an increase in neuronal density and neurite growth [52]. The glial scar is mostly composed of reactive astrocytes (VIM+/GFAP+) and often leads to failure in CNS regeneration [53].

Astrocyte proliferation (astrocytosis) is also a feature associated with neuroinflammation [54]. Previous studies have shown that inflammatory cytokines have the ability to induce astrocytosis. Tejada-Berges et al. showed that TNF-α could induce significant proliferation human primary adult astrocytes [55]. However, in that study astrocytes were grown in 15 to 30% FBS-containing medium whereas in our study the culture medium used for the proliferation assay was optimised at 5% serum (higher and lower concentrations were also tested - data not shown). In these conditions we did not observe any significant effect of TNF-α on astroglial proliferation. Two other studies have also reported that TNF-α could induce proliferation in human and simian adult astrocytes [56,57]. These discrepancies suggest a difference in TNF-α responsiveness between adult and foetal astrocytes. We then looked at IFN-γ ability to induce astrocytosis and we found, in accordance with previous studies, that this cytokine can significantly increase astrocyte proliferation. IFN-γ increases proliferation of human primary adult astrocytes in 5% FBS-containing culture medium [55] and murine neonatal astrocytes [47].

Finally, we assessed QUIN effect on astrocytosis. At low levels (150 nM) QUIN induces a significant increase of GFAP and proliferation of human astrocytes emphasizing that GFAP is a good marker for early stage astrocytosis. Astrocyte proliferation peaked in samples treated with pathophysiological concentration of QUIN (350) nM with a 20% increase in proliferation. This is in accordance with an earlier *in vivo *study showing that five days post-injection with QUIN (240 nM) in rat striatum, a significant increase in proliferation in GFAP-immunoreactive astrocytes could be observed [58]. Moreover, Martinez-Contreras et al have shown that glutamate-induced excitotoxicity can also display an increase in astrocyte proliferation, increased mRNA and protein expression of GFAP and VIM in rats [59]. At its highest pathological concentration QUIN (1200 nM) led to a significant reduction in cell proliferation, which is likely to be associated with cell death. We previously demonstrated that astrocytes were undergoing apoptosis when treated with QUIN 500 and 1200 nM [17].

Neuronal activity leads to the production of glutamate, which is then taken up and recycled by astrocytes [18]. Glutamine synthase (GS) is an enzyme playing a key role in the regulation of the glutamate-glutamine cycle within astrocytes. Decrease in GS activity has been previously reported in AD pathogenesis [19]. Tavares et al have shown that glutamate uptake by astrocytes is inhibited by QUIN [60]. A number of studies involving rat astrocytes and human gliomas have shown that changes in the glutamate uptake correlate with changes in GS activity [61,62]. Our end-point PCR results showed that GS is constitutively expressed even following QUIN or cytokine stimulations (Figure 1A). However, our functional study demonstrated that QUIN significantly inhibited GS activity in human foetal astrocytes in a dose dependent manner (Figure 1B). This latter finding supports observations from an earlier study showing that changes in GS activity are occurring at the protein level [19]. Some studies have shown that GS is sensitive to oxidative stress and free radicals that may induce structural change to the enzyme and leading to its inactivation [63,64]. Inhibition of GS activity by QUIN is likely that to be an indirect mechanism as this excitotoxin is known to induce the formation of free radicals [65]. Inhibition of GS by QUIN would lead to accumulation of glutamate in the extracellular environment, with enhanced excitotoxicity leading to neuronal death. Our data are in accordance with previous findings showing that astrogliosis is inversely correlated with a reduction in glutamine levels [7]. GS function is decreased in AD-affected regions while QUIN levels are increased [4,8] supporting further the involvement of QUIN in AD neuropathogenesis.

All together, this study suggests that QUIN is likely to play a key role in the complex neuropathogenesis of AD through several inflammatory and/or cytotoxic mechanisms. We showed that QUIN can induce IL-1β production, astrogliosis and inhibit GS function. These findings are likely to be relevant for the development of novel therapies for AD [66] and for several other neuroinflammatory diseases [2,67].

## Competing interests

The authors declare that they have no competing interests.

## Authors' contributions

KKT and GJG conceived and designed the experiments. KKT performed the experiments. KKT and GJG analyzed the data. BJB and GJG contributed reagents/materials/analytic tools. KKT and GJG wrote the paper. BJB helped with the writing and editing of the paper.

All of the authors have read and approved the final manuscript.
